# Supplementary material for: Socazolimab combined with carboplatin and etoposide as first-line treatment for extensive-stage small-cell lung cancer: A cost-effectiveness analysis in China
Source: PLoS One. 2025 Dec 22;20(12):e0339663. doi: 10.1371/journal.pone.0339663 (PMC12721548; doi:10.1371/journal.pone.0339663)
Supplement: Attachment — Submitted filename: Comments.docx [file pone.0339663.s001.docx]

Reviewer #1: Below are the comments/questions:
1. QALY Calculation & Utility Values: The model uses literature-derived utility values (PFS = 0.673, PD = 0.473), rather than trial-based utilities from NCT04878016. It also excludes disutilities for most immune-related AEs, including Grade 1–2 toxicities (e.g., hypothyroidism, pneumonitis), which may have a lasting QoL impact. Could the authors clarify why utilities were not collected prospectively in the trial? How do the selected values compare to other ICI studies in ES-SCLC (e.g., CASPIAN, IMpower133)?

Response: We thank the reviewer for this insightful comment and the opportunity to clarify our approach to utility values and the handling of adverse event (AE) disutilities. We agree that these are critical elements of the model.

1. Regarding the use of prospectively collected trial-based utility values.

This study did not utilize prospectively collected utility data from the target clinical trial (NCT04878016) primarily because the NCT04878016 trial did not directly measure or report utility values associated with the health states of PFS and PD. In pharmacoeconomic evaluations, when a pivotal clinical trial does not collect utility data, sourcing utility values from published literature specific to the same disease and health states is a widely accepted and standard methodological choice. This ensures the availability of model parameters and the feasibility of the analysis. Our approach is consistent with many published cost-effectiveness analyses in extensive-stage small cell lung cancer (ES-SCLC) [1-5].

2. Comparison of the selected utility values with other ES-SCLC immunotherapy studies (e.g., CASPIAN, IMpower133).

In fact, when selecting our utility values, we already referenced the common values used in the field of these specific studies to ensure the comparability and plausibility of our model parameters. The values for the PFS and PD health states were derived from a previously published study in SCLC, which has been cited by multiple Chinese pharmacoeconomic evaluations in lung cancer. We note that the PFS and PD utility values used in the cost-effectiveness analyses of the CASPIAN and IMpower133 studies are consistent with our data [3].

3. Regarding the exclusion of disutilities for most immune-related adverse events.

When constructing the model, to maintain model tractability and focus on the factors with the greatest impact on cost and effectiveness, we followed common practice in pharmacoeconomic modeling [5-7] by including only Grade ≥3 AEs with an incidence ≥5%. This is because these severe and common AEs are most likely to lead to significant healthcare resource utilization and short-term quality of life deterioration, thereby having a substantive impact on the cost-utility analysis results. While Grade 1-2 irAEs such as hypothyroidism and pneumonitis may have potential long-term effects, their severity, duration, and impact on overall quality of life vary considerably among patients, making them difficult to quantify precisely. Including them in the model would introduce significant uncertainty.

We sincerely thank you for your insightful comments. These limitations are indeed areas for potential improvement in our future research.

1. You M, Luo L, Lu T, et al. Cost-effectiveness analysis of benmelstobart, anlotinib, and chemotherapy in extensive-stage small-cell lung cancer. Front Immunol. 2024 Nov 25;15:1477146.
2. Long R, Chen F. First-line chemotherapy with tislelizumab for patients with extensive-stage small cell lung cancer: a cost-effectiveness analysis. Sci Rep. 2024 Dec 30;14(1):31958.
3. Ionova Y, Vuong W, Sandoval O, et al. Cost-Effectiveness Analysis of Atezolizumab Versus Durvalumab as First-Line Treatment of Extensive-Stage Small-Cell Lung Cancer in the USA. Clin Drug Investig. 2022 Jun;42(6):491-500.

4. Liang X, Chen X, Li H, et al. Cost-effectiveness analysis of first-line serplulimab combined with chemotherapy for extensive-stage small cell lung cancer. Front Public Health. 2023 Aug 31;11:1156427.

5. You M, Chen R, Wu Q, et al. Cost-effectiveness analysis of adebrelimab combined with chemotherapy for extensive-stage small cell lung cancer. Front Pharmacol. 2022 Oct 26;13:1019826.

6. Zhu Y, Liu K, Qin Q, et al. Serplulimab plus chemotherapy as first-line treatment for extensive-stage small-cell lung cancer: A cost-effectiveness analysis. Front Immunol. 2023 Jan 4;13:1044678.

7. Zhu Y, Liu K, Yang Q, Zeng M, Peng L. First-line Immuno-chemotherapy for extensive-stage small-cell lung cancer: A network meta-analysis and cost-effectiveness analysis. Front Public Health. 2023 Mar 16;11:1028202.
2. Survival Extrapolation: Long-term survival extrapolation introduces uncertainty, as acknowledged by the authors. The log-logistic model was selected based on AIC/BIC, but it's unclear whether goodness-of-fit was visually assessed (e.g., KM overlay, residuals). Why was the log-logistic model preferred over alternatives like Weibull or Gompertz, which are commonly used in oncology health economic models?

Response:

We thank the reviewer for this important comment regarding the selection and assessment of our survival extrapolation model. We agree that the choice of the long-term survival model is a critical source of uncertainty and warrants a thorough justification.

In our model, the selection of the survival distribution was based on a comprehensive approach that combined rigorous statistical criteria with graphical goodness-of-fit assessments. We fitted several parametric survival distributions commonly used in oncology health economic models, including the Exponential, Gompertz, Weibull, Log-logistic, and Lognormal distributions. The Akaike Information Criterion (AIC) and Bayesian Information Criterion (BIC) were calculated for each model. The results demonstrated that the Log-logistic model simultaneously yielded the lowest AIC and BIC values (see Table 1), providing strong statistical evidence that it was the best-fitting model for our dataset.

Furthermore, as the process of visual assessment was not clearly presented in the initial manuscript, we have now supplemented the manuscript with Kaplan-Meier (KM) overlay plots. As shown in Figures 1 and 2, the Log-logistic distribution curve demonstrates the closest alignment with the empirical KM curves within the observed data period (short-term fit). Regarding the long-term extrapolation, the "tail" effect predicted by the Log-logistic model is consistent with the clinical expectation of potential long-term survival benefits following immune checkpoint inhibitor treatment. In contrast, the Weibull and Gompertz distributions, which are frequently used in oncology economic models, showed a relatively greater deviation from the empirical data.

Table 1. AIC and BIC of survival curve in two groups

| Survival curve | Model criteria | Exponential | Gompertz | Weibull | Log-logistic | Lognormal |
| --- | --- | --- | --- | --- | --- | --- |
| Treatment group PFS curve  Control group  PFS curve  Treatment group OS curve  Control group  OS curve | AIC  BIC  AIC  BIC  AIC  BIC  AIC  BIC | 843.465  846.979  819.229  822.742  1342.252  1345.765  1394.877  1398.391 | 838.231  845.258  782.680  789.707  1282.369  1289.396  1323.131  1330.158 | 797.499  804.526  718.929  725.956  1258.094  1265.121  1294.772  1301.799 | 733.288  740.315  659.669  666.696  1251.844  1258.870  1286.559  1293.585 | 744.971  751.998  681.126  688.153  1255.642  1262.668  1297.517  1304.544 |

Figure 1

Figure 2

3. Cost Assumptions: The model adopts a healthcare system perspective and excludes indirect costs (e.g., productivity loss, caregiver burden), which may underestimate societal burden. Drug prices are based on 2024 bid prices (e.g., socazolimab at 115 CNY/mg). How would inclusion in China’s NRDL or price negotiations impact cost-effectiveness? Was a scenario analysis conducted with lower acquisition costs?
Response: We thank the reviewer for these insightful questions regarding our cost assumptions and their real-world implications.

This study adopts the healthcare system perspective primarily in accordance with the recommendations of the Chinese Guidelines for Pharmacoeconomic Evaluation. The guidelines recommend prioritizing this perspective for base-case analyses, thereby ensuring direct comparability of our findings with most domestic pharmacoeconomic evaluations [1-3] and facilitating reference for healthcare policy decision-makers. As per the guidelines, indirect costs (such as productivity losses and caregiver burdens) fall outside the direct responsibility of the healthcare system and represent broader societal economic impacts, which are less relevant to short-term decision-making within the healthcare system. Therefore, such costs are generally excluded under this perspective. In the 'Discussion' section, under study limitations, we explicitly address the constraints of adopting the healthcare system perspective and briefly explore the potential implications of incorporating indirect costs as follows:

“ Besides, our cost analysis was confined to direct medical costs and did not include indirect costs (such as productivity losses and caregiver burden). From a societal perspective, our study may underestimate the true economic burden of extensive-stage small cell lung cancer. Future research should aim to adopt a societal perspective by prospectively collecting data on productivity losses and caregiver burden to provide a more comprehensive economic evaluation”

In the one-way sensitivity analysis, the price of Socazolimab was identified as one of the most critical drivers influencing the Incremental Cost-Effectiveness Ratio (ICER). Therefore, we supplemented the analysis with a price simulation scenario assuming reduced procurement costs, simulating potential outcomes following national drug reimbursement negotiations. We set a series of hypothetical drug price discount rates (e.g., assuming price reductions of 30%, 50%, and 70%) and recalculated the corresponding ICERs (Table 2). The price simulation results demonstrated that when the price of Socazolimab was reduced by 30%, 50%, and 70%, the corresponding ICERs were 293,555.90 CNY/QALY, 252,381.90 CNY/QALY, and 211,207.90 CNY/QALY, respectively. When the price of Socazolimab was reduced by 50%, the ICER fell below the willingness-to-pay (WTP) threshold of 268,074 CNY, indicating that the Socazolimab combination regimen became cost-effective at this price point. This strongly suggests that the cost-benefit outlook of this regimen is closely linked to the outcomes of future price negotiations, and that price negotiation is a critical pathway to achieving its accessibility within China's healthcare system.

Table 2. ICER values at different discount rates for Socazolimab prices

| Price discount rate | Group | Total cost/  CNY | Incremental cost/CNY | Effect/  QALYs | Incremental effect/QALYs | ICER, CNY/QALY |
| --- | --- | --- | --- | --- | --- | --- |
| 70% | Treatment group | 128739.72 | 61646.74 | 0.87 | 0.21 | 293555.90 |
| 50% | Treatment group | 120093.18 | 53000.2 | 0.87 | 0.21 | 252381.90 |
| 30% | Treatment group | 111446.64 | 44353.66 | 0.87 | 0.21 | 211207.90 |
|  | Control group | 67092.98 |  | 0.66 |  |  |

1. Liu Q, Tan C, Yi L, et al. Cost-effectiveness analysis of pembrolizumab plus chemotherapy as first-line therapy for extensive-stage small-cell lung cancer. PLoS One. 2021 Nov 15;16(11):e0258605.
2. Zheng Z, Zhu H, Fang L. Cost-effectiveness analysis of adebrelimab in combination with chemotherapy for first-line treatment of extensive-stage small cell lung cancer. PLoS One. 2025 Jun 13;20(6):e0325171.
3. You C, Zhang J, Lei J, et al. Cost-effectiveness analysis of anlotinib plus chemotherapy with or without benmelstobart versus chemotherapy alone for extensive-stage small-cell lung cancer in China. Front Oncol. 2024 Dec 17;14:1484650.

4. Adverse Events & Model Scope: Only Grade ≥3 AEs with incidence ≥5% were modeled, potentially underestimating the true economic and QoL impact of chronic immune-related AEs (e.g., hypothyroidism, adrenal insufficiency). Given the known safety profile of PD-L1 inhibitors, can the authors justify the exclusion of these lower-grade, but clinically significant, events?

Response: We thank the reviewer for this critical comment regarding the scope of adverse events (AEs) included in our model.

You rightly pointed out that chronic immune-related adverse events (irAEs) may have long-term impacts on patients' quality of life and medical costs, which is a aspect we thoroughly considered during our model design process. Our decision to include only grade ≥3 adverse events with an incidence ≥5% in the base-case analysis was primarily based on the following methodological considerations:

1. Adherence to the principles of model simplification and focus on key drivers. When constructing a partitioned survival model, it is essential to strike a balance between model complexity and precision. Our goal was to build a robust, interpretable model focused on the clinical events that have the greatest impact on cost and utility outcomes. Severe adverse events (grade ≥3 and incidence ≥5%), which typically lead to hospitalization, additional specialized interventions, high treatment costs, and significant short-term quality of life deterioration, are widely recognized as "key drivers" that substantially influence economic evaluation results. Focusing the model on these events helps maintain its simplicity and transparency.
2. Constraints of data availability and consistency. A core limitation of this study is that the target clinical trial (NCT04878016) did not report utility values associated with health states or specific adverse events. For low-grade (1-2) irAEs, obtaining reliable, China-population-specific disutility values is particularly challenging. Forcing the inclusion of these parameters would require relying on numerous uncertain assumptions, which could ultimately undermine the credibility of the model results.
3. Comparability with methodological approaches in similar studies. We noted that many published cost-effectiveness analyses of landmark clinical trials for first-line immunotherapy in ES-SCLC (such as CASPIAN and IMpower133) [1-4] also adopted a modeling strategy focusing on grade ≥3 adverse events with an incidence ≥5%, ensuring comparability across studies.

We fully agree that comprehensively capturing the long-term impact of irAEs is an area for refinement in future research. Therefore, we have added the following statement in the 'Discussion' section of the revised manuscript as follows:

“The exclusion of low-grade chronic immune-related adverse events (irAEs) from the model represents a limitation, as this may lead to an underestimation of the total treatment-related burden. However, it should be emphasized that this approach makes the conclusions of our base-case analysis more conservative and robust. Future research, upon obtaining more detailed patient-reported outcome data and long-term follow-up data, could conduct more refined analyses on this aspect.”

1. Ionova Y, Vuong W, Sandoval O, et al. Cost-Effectiveness Analysis of Atezolizumab Versus Durvalumab as First-Line Treatment of Extensive-Stage Small-Cell Lung Cancer in the USA. Clin Drug Investig. 2022 Jun;42(6):491-500.

2. You M, Chen R, Wu Q, et al. Cost-effectiveness analysis of adebrelimab combined with chemotherapy for extensive-stage small cell lung cancer. Front Pharmacol. 2022 Oct 26;13:1019826.

3. Zhu Y, Liu K, Qin Q, et al. Serplulimab plus chemotherapy as first-line treatment for extensive-stage small-cell lung cancer: A cost-effectiveness analysis. Front Immunol. 2023 Jan 4;13:1044678.

4. Zhu Y, Liu K, Yang Q, Zeng M, Peng L. First-line Immuno-chemotherapy for extensive-stage small-cell lung cancer: A network meta-analysis and cost-effectiveness analysis. Front Public Health. 2023 Mar 16;11:1028202.

5. Comparator Choice: The comparison is limited to chemotherapy alone, excluding globally recommended regimens such as atezolizumab or durvalumab combinations. This limits the generalizability of the findings, especially in global health technology assessments. Could the authors discuss why an active comparator was not included, and whether this may bias the ICER in favor of socazolimab?

Response: We thank the reviewer for raising this important point regarding the choice of comparator.

This study is a cost-effectiveness analysis based on a Phase III clinical trial (NCT04878016). In this trial, the control arm was set as carboplatin plus etoposide chemotherapy alone, in order to validate the absolute efficacy and safety of Socazolimab in combination with chemotherapy compared to traditional chemotherapy. Consequently, from the perspective of the Chinese healthcare system, we directly evaluated the "incremental" value of the Socazolimab regimen based on the results of this clinical trial. The primary reason for not directly comparing Socazolimab with atezolizumab or durvalumab in the base-case analysis is the current absence of randomized controlled trials that directly compare Socazolimab with atezolizumab or durvalumab in the first-line treatment of extensive-stage small cell lung cancer. We acknowledge that not including atezolizumab or durvalumab combination therapies as direct comparators is indeed a limitation of this study. In the future, once clinical data from head-to-head comparisons between Socazolimab and other PD-L1 inhibitors become available, it will be crucial to conduct more precise cost-effectiveness analyses to provide decision-makers with more comprehensive information.

6. Sensitivity Analyses: The PSA indicates only a 21.9% chance of cost-effectiveness at a WTP threshold of 287,391 CNY/QALY. However, no analyses were presented for alternative thresholds (e.g., 1×GDP or 3×GDP). Additionally, key drivers identified in one-way sensitivity analysis (e.g., PD utility, drug cost) suggest the model is sensitive to parameter variation. Were threshold analyses conducted to identify the price level at which socazolimab becomes cost-effective?

Response:

We thank the reviewer for this critical observation. In the base-case analysis of this study, the threshold of 287,391 CNY/QALY we adopted is precisely three times China's per capita GDP in 2024. This was already stated in the "Model structure" section: "The willingness-to-pay (WTP) threshold was established at three times China's per capita gross domestic product (GDP) in 2024, equating to 287,391 yuan/QALY." Following your suggestion, we have supplemented the analysis with alternative thresholds of 1×GDP and 2×GDP. The results indicate (Figures 3, 4) that at the 1×GDP threshold (95,797 CNY/QALY), the cost-effectiveness probability for the socazolimab group is 0%; at the 2×GDP threshold (191,594 CNY/QALY), the probability is 2.8%. This demonstrates that the cost-effectiveness probability of the Socazolimab regimen is highly sensitive to the chosen WTP threshold.

Furthermore, following your recommendation, a threshold analysis was conducted to determine the drug price level required for Socazolimab to achieve cost-effectiveness. By calculating the corresponding ICER values under different discount rates for the Socazolimab price, it was determined that cost-effectiveness begins when the unit price discount for Socazolimab reaches approximately 67%, as shown in Table 3. The following has been added to the price simulation analysis in the revised manuscript: "Our analysis indicates that for the Socazolimab combination regimen to be cost-effective at the 3×GDP threshold, its unit price needs to be reduced to approximately 77 CNY/mg." Additionally, the following has been added to the Discussion section: "The threshold analysis revealed that a price reduction from the current level is necessary for Socazolimab to achieve cost-effectiveness at China's WTP threshold of three times the per-capita GDP. This finding can provide an important quantitative reference for future national drug reimbursement negotiations

Figure 3

Figure 4

Table 3. ICER values at different discount rates for Socazolimab prices

| Price discount rate | Group | Total cost/  CNY | Incremental cost/CNY | Effect/  QALYs | Incremental effect/QALYs | ICER, CNY/QALY |
| --- | --- | --- | --- | --- | --- | --- |
| 90% | Treatment group | 137386.27 | 70293.29 | 0.87 | 0.21 | 334729.95 |
| 80% | Treatment group | 133062.99 | 65970.01 | 0.87 | 0.21 | 314142.90 |
| 70% | Treatment group | 128739.72 | 61646.74 | 0.87 | 0.21 | 293555.90 |
| 68% | Treatment group | 127875.07 | 60782.09 | 0.87 | 0.21 | 289438.52 |
| 67% | Treatment group | 127442.74 | 60349.76 | 0.87 | 0.21 | 287379.81 |
| 60% | Treatment group | 124416.45 | 57323.47 | 0.87 | 0.21 | 272968.90 |
|  | Control group | 67092.98 |  | 0.66 |  |  |

7. Clinical Trial Data Integration: The model is based on efficacy data from NCT04878016 (OS: 13.9 vs. 11.6 months), but no hazard ratios or p-values are reported. Was the trial statistically powered for OS or PFS? Without these, the robustness of treatment effect assumptions is unclear. Also, how does the observed effect size compare to existing ICI trials in ES-SCLC?

Response:

We thank the reviewer for this essential comment regarding the statistical robustness of the efficacy data and its contextualization.

Based on the published results from the NCT04878016 trial, we have added the following in the revised manuscript: "The study demonstrated that patients treated with socazolimab exhibited a significant improvement in overall survival (OS) compared to those in the placebo + EC group (13.90 months vs 11.58 months, hazard ratio for death, 0.799; 95% CI, 0.652–0.979; p = 0.0158)." The sample size calculation for NCT04878016 was based on overall survival as the primary endpoint.

We contextualized the efficacy of Socazolimab within the global development landscape of immune checkpoint inhibitors for extensive-stage small cell lung cancer. A comparative analysis of data from the three pivotal trials is presented in Table 4. The results indicate that the OS hazard ratio for Socazolimab (0.799) falls within a range similar to that of atezolizumab (0.70) and durvalumab (0.73), all demonstrating an approximate 20%-30% reduction in the risk of death. This confirms that the survival benefit offered by Socazolimab is comparable and consistent with the efficacy of established immune checkpoint inhibitors in ES-SCLC. Regarding the absolute benefit, the median OS extension in the Socazolimab group compared to the control group was 2.32 months (13.9 - 11.58), a value highly similar to the extensions observed in the aforementioned international studies (2.0 months for atezolizumab, 2.7 months for durvalumab). The OS improvement achieved statistical significance (p < 0.05) in all three trials. This further supports the rationale and robustness of utilizing this clinical data for our cost-effectiveness model.

Table 4. Comparison of therapeutic efficacy data among three groups of trials

| Trials | Median OS（treatment group vs.control group） | Hazard ratio | 95% CI | P value |
| --- | --- | --- | --- | --- |
| IMpower133[1] | 12.3vs.10.3 | 0.70 | 0.54-0.91 | 0.007 |
| CASPIAN[2] | 13.0vs. 10.3 | 0.73 | 0.59-0.91 | 0.0047 |
| NCT04878016 | 13.90vs.11.58 | 0.799 | 0.652-0.979 | 0.0158 |

1. Horn L, Mansfield AS, Szczęsna A, et al. First-Line Atezolizumab plus Chemotherapy in Extensive-Stage Small-Cell Lung Cancer. N Engl J Med. 2018 Dec 6;379(23):2220-2229.

2. Paz-Ares L, Dvorkin M, Chen Y, et al. Durvalumab plus platinum-etoposide versus platinum-etoposide in first-line treatment of extensive-stage small-cell lung cancer (CASPIAN): a randomised, controlled, open-label, phase 3 trial. Lancet. 2019 Nov 23;394(10212):1929-1939.

Reviewer #2: Comment 1: During the review, it was noted that this manuscript is similar to the published study "Cost effectiveness of socazolimab plus chemotherapy vs. standard chemotherapy for first line treatment of extensive stage small cell lung cancer: a U.S. and China perspective" (DOI: 10.1007/s12094-025-04035-4). Both studies are based on the same Phase III clinical trial (NCT04878016) and evaluate the cost-effectiveness of Socazolimab combination therapy from the perspective of the Chinese healthcare system, arriving at the same core conclusion: the regimen is not cost-effective at the current price. Besides, key methods such as study design, discount rate, and time horizon are largely identical. The novelty of this paper needs to be clarified.

Response:

We thank the reviewer for this crucial observation and for providing the opportunity to clarify the distinct and novel contributions of our manuscript relative to the cited published study (DOI: 10.1007/s12345-678-9).

We have thoroughly reviewed the published research you indicated (DOI: 10.1007/s12094-025-04035-4). While our study is based on the same Phase III clinical trial (NCT04878016) and reached a similar preliminary conclusion regarding cost-effectiveness within the Chinese context, it provides significant and valuable innovations in methodology and analytical depth, as elaborated below:

1. Our study employed a partitioned survival model. This model utilizes reconstructed individual patient data from Kaplan-Meier curves to directly simulate the proportion of patients in different health states over time, without requiring the assumption of constant transition probabilities between states. This approach offers greater flexibility in capturing the complex relationship between progression-free survival and overall survival when evaluating immuno-oncology therapies. In contrast, the Markov model relies on estimating monthly transition probabilities between health states. The use of different model structures for cross-validation enhances the robustness and reliability of the cost-effectiveness analysis results.
2. Deepened Analytical Focus and Policy Implications: Analysis of Heterogeneous Willingness-to-Pay Across Chinese Regions. Our study conducted a more in-depth analysis that is better aligned with China's specific context. We recognize that China, as a large country with uneven development, lacks a unified national WTP threshold. Therefore, we specifically explored the feasibility of setting differentiated WTP thresholds based on the economic development levels of different regions (e.g., per capita GDP) in the Discussion section. We analyzed how the socazolimab combination regimen could potentially be cost-effective in more economically developed regions of China (such as Beijing, Shanghai, and Guangdong) if higher regional WTP thresholds were applied.

Consequently, although both studies indicate that the regimen is not cost-effective at the current price, our research provides new methodological evidence based on the partitioned survival model, deepens the discussion on China's heterogeneous payment capacity, and offers more nuanced insights for local healthcare decision-making and corporate market strategies in terms of policy implications. Thus, this study constitutes a valuable and novel supplement to the existing literature. To more clearly demonstrate this novelty, we have added a new paragraph in the Discussion section of the revised manuscript, systematically comparing the similarities and differences between our findings and the published study, and emphasizing the unique value and policy significance of our research:

“Although a cost-effectiveness analysis based on the same Phase III clinical trial (NCT04878016) has been published, our study employs a distinct modeling approach—the partitioned survival model (PSM). The PSM utilizes reconstructed individual patient data from Kaplan-Meier curves to directly simulate the proportion of patients in different health states over time, without requiring the assumption of constant transition probabilities between states. This approach offers greater flexibility in capturing the complex relationship between progression-free survival and overall survival when evaluating immuno-oncology therapies. In contrast, the Markov model used in the published study relies on estimating monthly transition probabilities between health states. While the core clinical findings are consistent, the use of different model structures for cross-validation strengthens the robustness and reliability of the cost-effectiveness analysis results. In addition, a key novel aspect of our analysis is the exploration of variable WTP thresholds reflective of China's significant regional economic disparities. Unlike a one-size-fits-all approach, our findings provide tailored insights for policymakers in more affluent provinces”。

Once again, we thank you for this valuable opportunity to improve the quality of our manuscript.

Comment 2: The utility values for the PFS and PD states (0.673 and 0.473) used in the study are cited from published literature. The authors need to clarify in the Discussion section whether these values were derived from a Chinese ES-SCLC population. If they were transferred from studies in other countries or different cancer types (e.g., non-small cell lung cancer), the applicability to the Chinese ES-SCLC patient population must be justified.

Response:

We thank the reviewer for raising this critical point regarding the applicability of the health state utilities to the Chinese ES-SCLC population.

The health utility weights used in this study (PFS state: 0.673, PD state: 0.473) were not directly sourced from a specific study targeting a Chinese ES-SCLC population. These values were derived from a published study investigating health utilities in non-small cell lung cancer patients receiving immunotherapy [1]. Despite the differences in population and cancer type, we selected these utility weights based on the following rationale:

1. Lack of China-specific data: Currently, there are no prospectively measured health utility values specifically for a Chinese ES-SCLC population reported in the publicly available literature. In the absence of ideal data, utilizing disease state-specific utility values from high-quality international studies, supplemented by extensive sensitivity analyses, is methodologically sound and aligns with health economic evaluation guidelines.
2. Similarity in Disease State and Symptom Burden: Patients with ES-SCLC and NSCLC experience comparable symptom burdens and treatment-related side effects within similar disease states. Particularly in the PD state, overall quality of life shows a substantial and common decline due to disease progression, regardless of the primary cancer type. Consequently, borrowing disease state-based utility weights across cancer types is a common and widely accepted practice in health economic evaluations, especially when country-specific data are missing. We reviewed other economic evaluations of ES-SCLC conducted in the Chinese context [2-6] and found that many studies, facing the same data gap, adopted a similar strategy by citing validated utility weights from the international literature. Our approach is consistent with the research practices in this field.
3. Addressing Uncertainty and Validating Robustness: To address this uncertainty and validate the robustness of our conclusions, the study incorporated comprehensive sensitivity analyses:

One-Way Sensitivity Analysis: As shown in the results, the utility values for PFS and PD were among the most influential parameters on the ICER. In the analysis, we tested a wide range of variation (±20%), and the results demonstrated that our primary conclusions remained stable even under such substantial variations.

Probabilistic Sensitivity Analysis (PSA): This analysis incorporated uncertainty for all parameters, including the PFS and PD utility values. The PSA results indicated that at the WTP threshold of three times the per-capita GDP (287,391 CNY/QALY), the probability of the Socazolimab regimen being cost-effective was only 21.4%. This low probability suggests that, even when potential variations in utility values for the Chinese population are fully considered, the current Socazolimab regimen is unlikely to be considered cost-effective at conventional thresholds. This significantly strengthens the robustness of our primary conclusion that the Socazolimab combination regimen is not cost-effective for the Chinese healthcare system at its current price.

1 Nafees B, Lloyd AJ, Dewilde S, et al. Health state Utilities in non-Small Cell Lung Cancer: an international study. Asia Pac J Clin Oncol. 2017;13:e195–203.

2.You M, Luo L, Lu T, et al. Cost-effectiveness analysis of benmelstobart, anlotinib, and chemotherapy in extensive-stage small-cell lung cancer. Front Immunol. 2024 Nov 25;15:1477146.

3.Long R, Chen F. First-line chemotherapy with tislelizumab for patients with extensive-stage small cell lung cancer: a cost-effectiveness analysis. Sci Rep. 2024 Dec 30;14(1):31958.

4. Ionova Y, Vuong W, Sandoval O, et al. Cost-Effectiveness Analysis of Atezolizumab Versus Durvalumab as First-Line Treatment of Extensive-Stage Small-Cell Lung Cancer in the USA. Clin Drug Investig. 2022 Jun;42(6):491-500.
5. Liang X, Chen X, Li H, et al. Cost-effectiveness analysis of first-line serplulimab combined with chemotherapy for extensive-stage small cell lung cancer. Front Public Health. 2023 Aug 31;11:1156427.
6. You M, Chen R, Wu Q, et al. Cost-effectiveness analysis of adebrelimab combined with chemotherapy for extensive-stage small cell lung cancer. Front Pharmacol. 2022 Oct 26;13:1019826.

Comment 3: A more in-depth explanation of the results of this study is required to clarify the value of this study.

Response: Thanks for your useful suggestion.

In response to your suggestion, we have made key revisions and additions to the Results section (specifically within the price simulation analysis) and the Discussion section of our manuscript. These additions focus on the analysis of a potential National Reimbursement Drug List (NRDL) negotiation price for Socazolimab, aiming to determine the specific drug price level required for it to achieve cost-effectiveness. This study directly provides an actionable, concrete price anchor. The analysis indicates that for Socazolimab to be cost-effective at a willingness-to-pay (WTP) threshold of three times the per-capita GDP, its unit price needs to be reduced to approximately 77 CNY/mg (equivalent to a 67% reduction from the current price). This offers a clear, quantified scientific basis for the upcoming NRDL negotiations. The quantitative analysis of the required price reduction holds significant policy implications for balancing patient accessibility, the sustainability of healthcare funds, and incentives for corporate innovation.

Comment 4: The authors mention regional economic disparities in China, stating that "for Beijing, Shanghai, Jiangsu, Fujian, Zhejiang, and Tianjin, the willingness-to-pay value is all greater than 397,500 yuan." However, this claim lacks citation. It is recommended to add relevant literature to support this statement.

Response: We thank the reviewer for this pertinent observation.

As recommended, we have supplemented the manuscript with the primary data source used to calculate the willingness-to-pay (WTP) thresholds for the involved provinces and cities. The 2024 per capita GDP data for all 31 provinces, autonomous regions, and municipalities directly under the central government in mainland China were obtained from the official website of the National Bureau of Statistics of China. The specific data used were sourced from: [https://data.stats.gov.cn/easyquery.htm?cn=C01](https://data.stats.gov.cn/easyquery.htm?cn=C01). The threefold values of the 2024 per capita GDP for the six mentioned regions exceeded the national average WTP threshold. In the revised manuscript, the official URL for the relevant data has been cited as a reference in 18, as follows:

18. National Bureau of Statistics of China. Regional Data: Gross Domestic Product [DB/OL]. [2025-03-01]. <https://data.stats.gov.cn/easyquery.htm?cn=C01.>
    Comment 5: On pages 13-14 (Results section), the concluding sentence states: "...the combination of siluximab and chemotherapy was not economically viable." Siluximab is an IL-6 inhibitor used for Castleman's disease. The authors must carefully check the entire manuscript.

Response:

Thank you for your thorough review of our manuscript and for identifying this error. Following your reminder, we have conducted a comprehensive and careful check of the entire text to ensure that all instances of the drug name have been corrected to "socazolimab."

Comment 6:Figures: it is advised to provide high-quality figures. Labels and format should be standardized.

Response: We thank the reviewer for this valuable feedback regarding the presentation quality of the figures.

As per your suggestion, we have conducted a comprehensive review and standardization of all figures and tables in the manuscript. The specific revisions are as follows:

In accordance with the journal's requirements, all figures have been regenerated as high-resolution versions. These high-quality image files have been uploaded separately as individual files in the submission system to ensure optimal clarity and professional presentation upon publication.

We have unified and standardized the labels (including font, font size, and capitalization format) and legends for all figures and tables to ensure consistency throughout the manuscript. Detailed captions have been added to every table in the manuscript. All table captions are placed below their respective tables and clearly explain the abbreviations used and other key information requiring clarification. The specific details are as follows:

Table 1. AIC and BIC of survival curve in two groups

| survival curve | Model criteria | Exponential | Gompertz | Weibull | Log-logistic | Lognormal |
| --- | --- | --- | --- | --- | --- | --- |
| Treatment group PFS curve  Control group  PFS curve  Treatment group OS curve  Control group  OS curve | AIC  BIC  AIC  BIC  AIC  BIC  AIC  BIC | 843.465  846.979  819.229  822.742  1342.252  1345.765  1394.877  1398.391 | 838.231  845.258  782.680  789.707  1282.369  1289.396  1323.131  1330.158 | 797.499  804.526  718.929  725.956  1258.094  1265.121  1294.772  1301.799 | 733.288  740.315  659.669  666.696  1251.844  1258.870  1286.559  1293.585 | 744.971  751.998  681.126  688.153  1255.642  1262.668  1297.517  1304.544 |

AIC: Akaike information criterion, BIC: Bayesian information criterion, PFS: progression-free survival, OS: overall survival.

Table 2. Parameter distribution of survival curve in two groups

| Survival curve | Optimal fitting distribution | Shape | Scale |
| --- | --- | --- | --- |
| Treatment group PFS curve  Control group PFS curve  Treatment group OS curve  Control group OS curve | Log-logistic  Log-logistic  Log-logistic  Log-logistic | 3.07673  3.97539  2.63812  2.83146 | 6.88847  5.39080  13.97048  12.47755 |

PFS: progression-free survival, OS: overall survival.

Table 3. Model Parameters

| Variable | Baseline Value | Minimum | Maximum | Distribution | Reference |
| --- | --- | --- | --- | --- | --- |
| Cost (CNY) |  |  |  |  |  |
| Socazolimab/mg | 115 | 92 | 138 | Gamma | https://www.yaozh.com/ |
| Etoposide/mg | 3.16 | 2.53 | 3.80 | Gamma | https://www.yaozh.com/ |
| Carboplatin/mg | 0.61 | 0.49 | 0.73 | Gamma | https://www.yaozh.com/ |
| Laboratory testing | 166 | 132.8 | 199.2 | Gamma | [20] |
| Radiological examinations | 300 | 240 | 360 | Gamma | [21] |
| Anemia | 1235.30 | 988.24 | 1482.36 | Gamma | [22] |
| Leukocytopenia | 2309.99 | 1847.99 | 2771.99 | Gamma | [23] |
| Decreased neutrophil count | 2877.40 | 2301.92 | 3452.88 | Gamma | [23] |
| Decreased platelet count | 822.89 | 658.31 | 987.47 | Gamma | [22] |
| BSC | 3115 | 2492 | 3738 | Gamma | [24] |
| Incidence rate of adverse reaction/% |  |  |  |  |  |
| Decreased neutrophil count (Treatment group) | 69.1 | 55.28 | 82.92 | Beta | [8] |
| Leukocytopenia (Treatment group) | 44.2 | 35.36 | 53.04 | Beta | [8] |
| Anemia (Treatment group) | 23.3 | 18.64 | 27.96 | Beta | [8] |
| Decreased platelet count (Treatment group) | 33.7 | 26.96 | 40.44 | Beta | [8] |
| Decreased neutrophil count (Control group) | 66.8 | 53.44 | 80.16 | Beta | [8] |
| Leukocytopenia (Control group) | 34.0 | 27.2 | 40.8 | Beta | [8] |
| Anemia (Control group) | 20.6 | 16.48 | 24.72 | Beta | [8] |
| Decreased platelet count (Control group) | 27.9 | 16.48 | 24.72 | Beta | [8] |
| PFS | 0.673 | 0.538 | 0.808 | Beta | [16-17] |
| PD | 0.473 | 0.378 | 0.568 | Beta | [16-17] |
| Discount rate/% | 5 | 0 | 8 | Beta | [12] |
| Weight | 59 | 47.2 | 70.8 | Normal | [13] |
| Body surface area/m^2^ | 1.72 | 1.38 | 2.06 | Normal | [14] |
| Creatinine clearance rate (ml/min) | 90 | 80 | 100 | Gamma | [15] |

BSC: best supportive treatment, PFS: progression-free survival, PD: progressive disease.

Table 4. Baseline Results

| Parameters | Treatment group | Control group |
| --- | --- | --- |
| Total cost/CNY  Incremental cost/CNY  Effect/QALYs  Incremental effect/QALYs  ICER, CNY/QALY | 141709.54  74616.56  0.87  0.21  355316.95 | 67092.98  0.66 |

CNY: chinese yuan, QALY: quality-adjusted life year, ICER: incremental cost-effectiveness ratio.

Comment 7:There are some mistakes in the reference format. Reference [8] is incomplete, containing only authors and the title, missing journal name, volume, issue, and page numbers. All references should be checked and completed to ensure the format conforms to PLOS ONE requirements.

Response:

Thank you for pointing this out. The following citation has been added to the revised manuscript:
Chen Z, Chen J, Huang D, et al. Socazolimab Study Group. A multicenter, randomized, double-blind, placebo-controlled phase 3 study of Socazolimab or placebo combined with carboplatin and etoposide in the first-line treatment of extensive-stage small cell lung cancer. Signal Transduct Target Ther. 2025;10(1):28.
