# Supplementary material for: Socazolimab combined with carboplatin and etoposide as first-line treatment for extensive-stage small-cell lung cancer: A cost-effectiveness analysis in China
Source: PLoS One. 2025 Dec 22;20(12):e0339663. doi: 10.1371/journal.pone.0339663 (PMC12721548; doi:10.1371/journal.pone.0339663)
Supplement: Attachment — Submitted filename: Response to Reviewers.docx [file pone.0339663.s004.docx]

**Only one minor revision is required prior to acceptance:**

**1. Figure 3 (Tornado Diagram): Please revise Figure 3 (tornado diagram) by replacing all abbreviated parameter names on the y-axis with full, descriptive labels.If you must retain any abbreviations due to space limitations, please spell out the full term in the figure caption (e.g., “u_pfs: utility in the progression-free survival state”). This change is required to ensure clarity for non-specialist readers and improve the figure’s interpretability.**

Response: Thanks for your advice. The requested revisions to Figure 3 have been completed, and the updated figure has been uploaded.

**Journal Requirements:**

**1. If the reviewer comments include a recommendation to cite specific previously published works, please review and evaluate these publications to determine whether they are relevant and should be cited. There is no requirement to cite these works unless the editor has indicated otherwise.**

Response: Thanks for your advice. In the first revision of the manuscript, the reviewer did not request the citation of any recommended literature. The references we added during the revision were based on the needs arising from our own modifications.
**2. Please review your reference list to ensure that it is complete and correct. If you have cited papers that have been retracted, please include the rationale for doing so in the manuscript text, or remove these references and replace them with relevant current references. Any changes to the reference list should be mentioned in the rebuttal letter that accompanies your revised manuscript. If you need to cite a retracted article, indicate the article’s retracted status in the References list and also include a citation and full reference for the** retraction notice.

Response: Thanks for your suggestion. All references were complete and correct.
